# Comprehending the Role of Metabolic and Hemodynamic Factors Alongside Different Signaling Pathways in the Pathogenesis of Diabetic Nephropathy

**DOI:** 10.3390/ijms26073330

**Published:** 2025-04-03

**Authors:** Yashumati Ratan, Aishwarya Rajput, Ashutosh Pareek, Aaushi Pareek, Gurjit Singh

**Affiliations:** 1 Department of Pharmacy, Banasthali Vidyapith, Banasthali 304022, Rajasthan, India; yashu.bu@gmail.com (Y.R.); kailashaish@gmail.com (A.R.); ashu83aadi@gmail.com (A.P.); aaushipareek26@gmail.com (A.P.); 2 Pantheravax LLC, Ames, IA 50011, USA

**Keywords:** diabetic nephropathy, diabetic kidney disease, molecular mechanism, pathogenesis, metabolic pathways, hemodynamic pathways

## Abstract

Diabetic nephropathy (DN) is a progressive microvascular disorder of diabetes that contributes as a primary reason for end-stage renal disease worldwide. The pathological hallmarks of DN include diffuse mesangial expansion, thicker basement membrane of glomeruli, and arteriole hyalinosis. Hypertension and chronic hyperglycemia are the primary risk factors contributing to the occurrence of DN. The complex pathophysiology of DN involves the interplay amongst metabolic and hemodynamic pathways, growth factors and cytokines production, oxidative stress, and ultimately impaired kidney function. Hyperglycemia-induced vascular dysfunction is the main pathological mechanism that initiates DN. However, several other pathogenic mechanisms, such as oxidative stress, inflammatory cell infiltration, and fibrosis, contribute to disease progression. Different vasoactive hormone processes, including endothelin and renin–angiotensin, are activated as a part of the pathophysiology of DN, which also involves increased intraglomerular and systemic pressure. The pathophysiology of DN will continue to be better understood because of recent developments in genomics and molecular biology, but attempts to develop a comprehensive theory that explains all existing cellular and biochemical pathways have been thwarted by the disease’s multifactorial nature. This review extensively discusses the current understanding regarding the metabolic and hemodynamic pathological mechanisms, along with other signaling pathways and molecules responsible for the pathogenesis of DN. This work will encourage a greater in-depth understanding and investigation of the present status of the biochemical mechanistic processes underlying the pathogenesis of DN, which may assist in the determination of different biomarkers and help in the design and development of novel drug candidates in the near future.

## 1. Introduction

**Figure 1:**
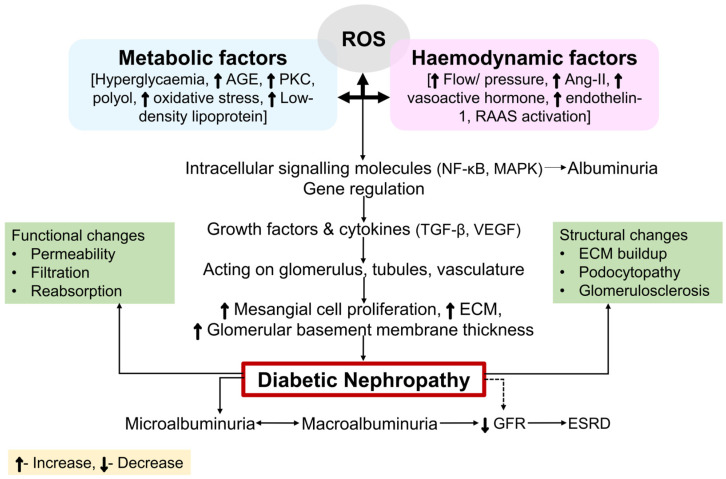
An illustration of how intracellular signaling molecules and cytokines, alongside the metabolic and hemodynamic pathways, interplay to cause DN. DN is believed to develop because of conjugation between hemodynamic and metabolic aberrancies that interact with multiple pathways dependent upon ROS and each other. The interplay of hemodynamic variables, metabolic stimulation, and ROS formation affects not just TF activation but also gene regulation. The characteristic symptoms of DN are brought on by structural and functional alterations resulting from this molecular activation or inhibition. Abbreviations: ROS, reactive oxygen species; AGE, advanced glycation end products; PKC, protein kinase C; Ang-II, angiotensin-II; RAAS, renin angiotensin aldosterone system; NF-κB, nuclear factor-κB; MAPK, mitogen-activated protein kinase; TGF-β, transforming growth factor-β; VEGF, vascular endothelial growth factor; ECM, extracellular matrix; GFR, glomerular filtration rate; ESRD, end-stage renal disease.

Diabetes mellitus (DM) is a metabolic disease characterized by hyperglycemia, resulting from either insufficient insulin secretion, an impairment in insulin functionality, or both. Type 1 diabetes mellitus (T1DM) is a prevalent form of DM, which displays an absolute dearth of insulin because of damage to the beta cells in the pancreas. On the other hand, insulin resistance is the cause of hyperglycemia in type 2 diabetes mellitus (T2DM) [1]. T2DM is the leading cause of diabetes-induced death worldwide because of its high prevalence and serious complications, which include diabetic neuropathic pain and kidney and cardiovascular disorders that can result in early deaths [2,3,4]. Diabetic nephropathy (DN) is one of the primary microvascular complications of DM, which affects around 40% of people with DM. The decline of kidney function observed in chronic T1DM and T2DM patients is known as DN or diabetic kidney disease (DKD) [5]. It is characterized by the onset of proteinuria, which is accompanied by a declined glomerular filtration rate (GFR) and an increase in blood pressure (BP) in the arteries [6,7]. DN is the most prevalent cause of chronic kidney disease (CKD) morbidity, end-stage renal disease (ESRD), and mortality globally, and it substantially undermines patient safety, health, and standard of living [8,9]. T1DM and T2DM are both marked by persistently higher blood sugar concentrations, which is the major cause of DN. High blood sugar causes the renal blood vessels to deteriorate, which sets off a series of events that culminate in kidney failure [10]. Hypertension, genetic susceptibility, smoking, obesity, elevated cholesterol, and proteinuria are the main causes of DN [11]. The signs and symptoms seem to be non-existent at the initial stage. Impaired BP control, proteinuria, swelling in the hands, feet, or eyes, elevated urogenital urges, decreased need for insulin or diabetic drugs, disorientation or difficulty concentrating, dyspnea, diminished appetite, nausea and vomiting, chronic itching, and tiredness are some of the signs and symptoms of the advanced stages [12,13]. Given recent findings, DN is thought to be caused by abnormalities in important cellular components and processes that facilitate renal failure. The pathophysiology of DN is interlinked with several factors, including increased glucose metabolite flow [10], increased glycation end products [14], mitochondrial (Mt) dysfunction [15], endoplasmic reticulum stress [16], a hyperactive renin–angiotensin–aldosterone system (RAAS) or renin–angiotensin system (RAS), and oxidative stress [17]. DN results from correlations between metabolic and hemodynamic factors, as shown in Figure 1 [18]. Hemodynamic variables that contribute to the development of DN include high intraglomerular and systemic strain and several active vasoactive hormone pathways, such as RAS and endothelin [19]. These hemodynamic pathways stimulate nuclear transcription factors like vascular endothelial growth factor (VEGF) and nuclear factor-κB (NF-κB), along with intracellenic secondary messengers like protein kinase C (PKC) and mitogen-activated protein kinase (MAPK). Advanced glycation end product (AGE) aggregation, renal polyol production, and enhanced oxidative stress are all caused by glucose-related mechanisms, which are most frequently activated in the diabetic kidney. Furthermore, these mechanisms lead to enhanced glomerulosclerosis (GMS), proteinuria, and tubulointerstitial fibrosis by gradually improving the permeation of renal albumins and the extracellular matrix (ECM) [20]. The purpose of this work is to elucidate a clear picture of the molecular mechanistic pathways behind the etiopathogenesis of DN, which would further facilitate the design and development of novel mechanism-based therapeutics for DN.

## 2. Epidemiology and Prevalence of DN

DM has grown into a considerable worldwide community health concern. Globally, both its incidence and prevalence have risen. In correspondence to the “International Diabetes Federation”, around 537 million adults of 20–79 years of age suffer from DM [21]. This age bracket translates to 10.5% of the global population. It is predicted that, globally, DM subjects would increase from 643 million (11.3%) in 2030 to 783 million (12.2%) in 2045. Countries with low and moderate incomes account for three out of every four adult diabetics. Most people with DM (240 million) do not receive a diagnosis. T1DM affects more than 1.2 million kids and teenagers (ages 0 to 19). Diabetes during pregnancy affects one in six live births (21 million). Additionally, 541 million persons have a higher chance of getting T2DM [21]. DKD will eventually occur in 20–50% of individuals with T2DM. With DKD contributing to 50% of cases globally, it is the primary cause of ESRD and CKD [22].

Multicentred research conducted in India estimated the prevalence of DKD to be approximately 62.3% [21,22]. A cross-sectional study in China found that 38.8% of 15,856 diabetic individuals had CKD [23]. After a nine-year monitoring period, an epidemiological investigation from the United Arab Emirates showed a cumulative incidence of CKD of 11.4% [24]. The Eastern Mediterranean region’s elderly population exhibited the highest prevalence rates of DKD and DM [25]. Owing to a survey of findings from the Global Burden of Disease Study 2019, 2.2% of US people had DKD. Furthermore, from 1988 to 2008, the US saw a corresponding upsurge in the prevalence of DKD and DM [26].

## 3. Risk Factors of DN

Per the claims of the findings from multiple epidemiological research studies, the primary risk factors for DN are dyslipidemia, gestational DM, insulin resistance, high BP, ethnicity, obesity, and family history [27]. Smoking, increased systolic pressure, raised glycosylated haemoglobin (HbA1c) percentage, and proteinuria are other potential risk factors [27]. Nephropathy is the most reliable prognostic factor of death in diabetic patients, yet there are significant inter-individual differences in its development. High-throughput techniques and genome-wide transcriptome investigations show that inflammatory signaling pathways and oxidative stress are activated, emphasizing the significance of genetic variables [28,29]. Research suggests that epigenetic processes such as histone changes, noncoding ribonucleic acid (RNA), and deoxyribonucleic acid (DNA) methylation might be crucial in DN development. Thus, polymorphisms in the promoter regions of inflammatory cytokines, including tumor necrosis factor-α, interleukin-6, and interleukin-1β, along with changes in their production, have been connected to DN susceptibility in patients. Kidney injury may be caused by tissue remodeling that follows inflammation-induced dysregulation of the local metabolic system [30]. Numerous signaling pathways, including the polyol pathway, diacylglycerol (DAG)-PKC route, hexosamine pathway, AGE system, and oxidative stress, have been demonstrated to be triggered by excessive intracellular glucose [31].

### 3.1. Age and Race 

Individuals diagnosed with T2DM before turning 20 years old are more likely to get severe renal failure [32]. African American, Asian Indian, and Mexican American ethnic groups have increased rates of DN compared to other populations, primarily due to a higher prevalence of diabetes within these communities, which makes them more susceptible to complications like kidney damage from diabetes [22].

### 3.2. Obesity

It is uncertain exactly how fat contributes to diabetic kidney damage. Obesity-related glomerulopathy is thought to be caused by obesity alone, unaffected by glomerular injury, proteinuria, hyperglycaemia, and glomerular hypertrophy [33].

### 3.3. Gene Susceptibility

Genes encoding the cytokines, angiotensin-converting enzyme (ACE), proteins, and angiotensin-II (Ang-II) receptor participate in the metabolism of fats, carbohydrates, and ECM proteins [33]. Nephropathy associated with DM is correlated with chromosomes [22,34,35].

### 3.4. Duration of DM

The greatest risk factor associated with nephropathy is having diabetes for a longer period. Nephropathy can result in elevated levels of HbA1c, polyol pathway activation, and AGE [36].

### 3.5. Hypertension

Hypertensive nephropathy, alongside other residing risk factors, may cause CKD in individuals with DM [37].

### 3.6. Raised Albuminuria

Elevated albumin excretion in urine occurs, which is known as macroalbuminuria (>300 mg/g) or microalbuminuria (30–300 mg/g) [38].

### 3.7. Dyslipidemia

The “lipid nephrotoxicity hypothesis” outlines how dyslipidemia can negatively impact kidney functioning. It seems that the occurrence of DKD is mostly influenced by macrophage infiltration, increased ECM formation, and podocyte mortality [21,39].

### 3.8. Smoking

Smoking also plays a part in DN development through a variety of pathogenic mechanistic processes like oxidative stress, AGE accumulation, hyperlipidemia, and GMS [34].

## 4. Progression of DM into DN

**Figure 2:**
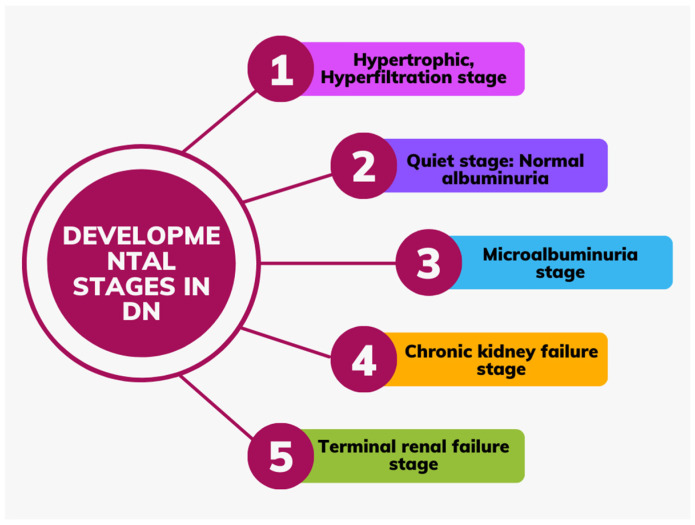
Stages of development in DN.

Microalbuminuria is considered the first sign of nephropathy in clinical terms. Five to fifteen years after diagnosis, it happens in thirty percent of people with T1DM. However, since the cause of T2DM is typically unclear, it can exist at the time of diagnosis. Figure 2 depicts the phases of DN development and progression. Despite being a useful diagnostic tool for DN, macroalbuminuria is not always the outcome for all patients with microalbuminuria. Indeed, according to some patients, normoalbuminuria may relapse [40]. BP and blood sugar control levels appear to impact the unpredictable course of renal disease in T1DM subjects. As a result, although early research indicated that over 80% of patients with microalbuminuria advance to proteinuria over the course of 6–14 years, more recent research has shown a regression due to improved glycaemic control [41,42,43,44]. After around ten years, ESRD is reached when overt proteinuria starts to deteriorate renal function. Patients with T1DM also had an 89% lower chance of experiencing a decline in GFR when their microalbuminuria improved [45,46]. Since kidney disease in T2DM subjects is typically diagnosed alongside another illness, its progression and regression are very unpredictable [47,48]. According to epidemiological research conducted on Indians, the frequency of overt nephropathy varies based only on the length of time since the onset of the disease, ranging from 21% for people with T1DM to 20–25% for individuals with T2DM.

## 5. Developmental Stages in DN

The five main stages of DN development are depicted in Figure 2. Once the disease starts, stage 1 lasts for around five years, during which time the GFR is neither normal nor high. Even while enhanced elimination of albumin and arterial pressure remains within normal limits, renal dimensions increase by about 20%, and kidney blood circulation jumps by 10–15%. Around 2 years following disease onset, the second stage is marked by the destruction of the kidneys, proliferating mesangial cells (MCs), and thickened basement membrane. There are currently no symptoms that would suggest the condition. GFR levels revert to normal. Stage 3 is the beginning stage of nephropathy, with an albumin range of 30–300 mg/dU. It typically manifests 5–10 years after the onset. This is the first clinically observable sign of glomerular damage. The arterial pressure may be normal or high. Usually, about 40% of patients advance to this stage. The irreversible stage 4 is characterized by elevated BP beyond normal, a drop in GFR to less than 60 mL/min, and the development of proteinuria (albumin > 300 mg/dU). In stage 5, the GFR drops to less than 15 mL/min. About 50% of patients with terminal renal failure require kidney replacement therapy, which includes kidney transplantation, hemodialysis, and peritoneal dialysis [49].

## 6. Molecular Mechanisms in the Pathogenesis of DN

The pathophysiology of DN is characterized by the convoluted and multifaceted molecular mechanisms that involve multiple pathways and mediators alongside interrelated metabolic and hemodynamic variables [50]. Figure 1 depicts the molecular relationships of the intracellular signaling components, cytokines, and hemodynamic and metabolic pathways that underlie the pathogenesis of DN. Using several types of mediators, each pathway either causes destruction or engages with distinct pathways. Amongst the mediators and different pathways, there exists a tremendous degree of crossover. For example, Ang-II damages cells through oxidative stress, which further induces abnormalities via RAAS. Nicotinamide adenine dinucleotide phosphate hydrogen (NADPH) oxidase stimulates transforming growth factor-β (TGF-β), which further induces ROS via activating NADPH oxidase (Nox) [51,52]. This means that the exact patho-mechanisms and molecular development of DN remain poorly understood, and the relative contributions of every pathway towards the emergence of DN are undefined.

### 6.1. Metabolic Mechanisms

#### 6.1.1. Polyol Pathway

**Figure 3:**
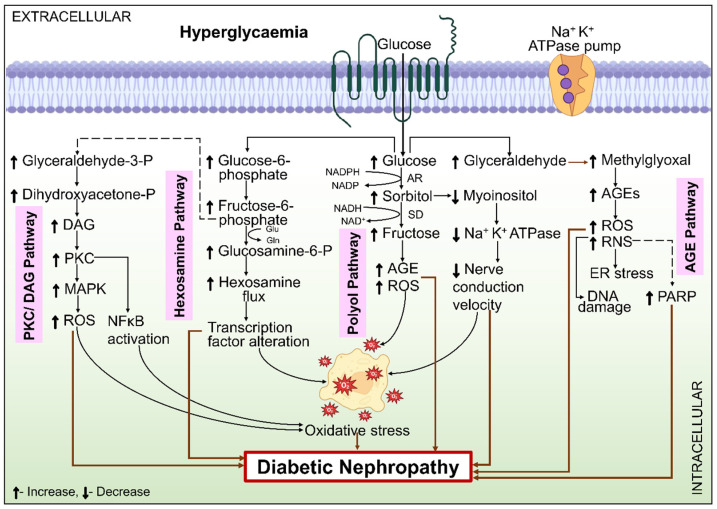
A systematic representation of different metabolic pathways in the pathophysiology of DN. When combined with oxidative stress, hyperglycemia sets off the harmful pathways of polyol, PKC, AGE, and hexosamine. This results in redox imbalance, changes in gene expression, and altered transcription factors, all of which intensify oxidative stress and eventually cause DN. Abbreviations: DAG, diacylglycerol; PKC, protein kinase C; MAPK, mitogen-activated protein kinase; ROS, reactive oxygen species; NF-κB, nuclear factor-κB; NADPH, nicotinamide adenine dinucleotide phosphate hydrogen; NADP, NADH, nicotinamide adenine dinucleotide hydrogen; nicotinamide adenine dinucleotide phosphate; NAD^+^, nicotinamide adenine dinucleotide; AR, aldose reductase; SD, soluble dehydrogenase; AGE, advanced glycation end products; RNS, reactive nitrogen species; ER, endoplasmic reticulum; DNA, deoxyribonucleic acid; PARP, poly ADP-ribose polymerase.

One of the bypassing metabolic processes of glucose, the polyol–aldose reductase pathway, is activated by hyperglycemia, which sets off a chain of metabolic events. There are two enzymes in it. Initially, aldose reductase (AR) is the enzyme that limits the rate. As more glucose is metabolized via this pathway in hyperglycemic conditions, AR activity rises [53]. This has several detrimental implications that exacerbate the development of DN. First, the membranes of the renal cells are harmed by the osmotic stress brought on by the buildup of intracellular sorbitol [54]. Soluble dehydrogenase (SD), the second enzyme, employs cofactor nicotinamide adenine dinucleotide (NAD)^+^ to obtain fructose from sorbitol, whereas AR needs cofactor NADPH to transform glucose into sorbitol. By raising the NADH/NAD ratio, this mechanism triggers oxidative stress and PKC [55], which in turn, activates TGF-β, triggering pathological consequences. Lastly, the byproducts of the polyol process, including sorbitol and fructose, act as highly potent glycosylating components compared to glucose by itself, resulting in elevated AGE [56]. NADPH contents are reduced as the outcome of AR NADPH consumption. Oxidized glutathione is transformed into reduced glutathione in the presence of NADPH, which is mandatory for the glutathione reductase action. Reduced glutathione is subsequently required by glutathione peroxidase. Eventually, this causes the antioxidant state to decline in the cell [57]. The polyol pathway in DN pathology is illustrated in Figure 3.

#### 6.1.2. Hexosamine Pathway

The biosynthesis of glycolipids, glycoproteins, and proteoglycans depends on the hexosamine pathway. The final product of the hexosamine process is UDP-N-acetyl-glucosamine, which is needed for the production of these compounds [58]. Hexokinase facilitates the conversion of glucose to glucose-6-phosphate, which is transformed into fructose-6-phosphate during the glycolytic cycle. Following glycolysis, the conversion of fructose-6-phosphate leads to the formation of proteoglycans and O-linked glycoproteins. Glutamine fructose-6-phosphate amido-transferase (GFAT) incorporates the amino donor glutamine to regulate this process, as depicted in Figure 3. Following that, several further reactions occur, causing the ultimate production of more glucosamines that work as a foundation for the amino sugars required to create proteoglycans, glycoproteins, and glycolipids. To circulate more readily, the hexosamine pathway requires higher TGF-1 expression and stronger PKC activation [59]. High glucose levels in cultured MCs cause the synthesis of TGF-β1, and GFAT inhibition reverses this effect. Conversely, the synthesis of TGF-β1 is increased when GFAT is overexpressed. Moreover, PKC appears to be the transducer for these effects. In human glomerular cells, GFAT is not present. However, GFAT expression in the glomerulus has been observed in individuals with DN, indicating that it might have a pathogenic function [60].

#### 6.1.3. PKC Pathway

PKC is a class of enzymes that are essential intracellular signaling components for blood vessel functioning. In the case of a normal body state, these enzymes are activated, and intracellular calcium ions and DAG are released via receptor-mediated PKC activation. Pathological conditions like DM might cause an aberrant increase in DAG synthesis, which can activate PKC. The pathological involvement of the PKC/DAG pathway in DN is presented in Figure 3. Enhanced glycolysis and higher glycerol-3-phosphate and glyceraldehyde-3-phosphate intracellular amounts cause DM and enhance DAG synthesis. Reaction to external stimuli, in turn, causes MAPK to become active. The co-activization of PKC and MAPK in the presence of elevated hyperglycemia suggests that DN continues to develop because of these two groups of enzymes [61]. PKC is activated by glucose, Ang-II, and AGE, which are found to be increased in the kidneys of diabetics. ROS can activate PKC. Controlling angiogenesis, cellular proliferation, leukocyte adhesion, vasoconstriction, cytokine activation, endothelial permeation, PKC activation, and ECM maintenance influences vascular functioning [62]. PKC activation has been observed to increase TGF-β production, which causes the ECM protein to accumulate and cause interstitial fibrosis [63]. Moreover, PKC has been demonstrated to play a part in controlling the expression of Nox2 and Nox4, as well as the production of ROS, which may be related to its effects [64]. In a phase 2 clinical trial, individuals with T2DM who have long-term albuminuria [“albumin-to-creatinine ratio” 200–2000 mg/g creatinine] after receiving RAS inhibitor medication were examined for an inhibitor of PKC-β, ruboxistaurin [65]. The main objective of the research, which was a reduction in the albumin-to-creatinine ratio after one year, was not statistically significant when compared with the placebo [66].

#### 6.1.4. AGE

The free amino acids present in plasma or tissue proteins are attached to excessive glucose resulting from chronic hyperglycemia. This non-enzymatic procedure ends up in the formation of irreversible AGE and reversible early glycated products in the tissues, which cause the microvascular difficulties associated with DM [61]. During the impact of oxidative stress and hyperglycemia, proteins, lipids, or DNA can suffer persistent structural alterations that result in the formation of AGE. Although HbA1c is an earlier glycation product, a similar mechanism generates HbA1c, which is utilized as a glycemic management marker and an indicator of the problems associated with DM. Over a cell lifetime, AGE buildup and AGE concentrations are correlated with DN rates in the kidney [67]. The pathogenic role of the AGE pathway in DN is shown in Figure 3.

Damage is caused by AGE through multiple mechanisms: they can stiffen the glomerular basement membrane and the walls of arteries by the cross-linkage of collagen in the ECM, affecting protein performance and turnover. Additionally, AGE can induce growth factors and profibrotic cytokines like TGF-β and VEGF through receptors for advanced glycation end products (RAGE) and encourage the formation of ROS from mitochondria and cytosolic sources, such as Nox [66,68]. The initiation of DN, which can be triggered by AGE, is significantly influenced by connective tissue growth factor 34 and TGF-β. In DN, RAGE and AGE have been investigated as possible targets. AGE can be present in typical foods like cheese and pork. However, there is not much proof that reducing dietary AGE is beneficial [69]. The B-group vitamins thiamine (B1) and pyridoxamine (B6) did not significantly affect DKD in clinical trials, despite appearing to show indications of lowering AGE in preclinical investigations [70,71]. Meanwhile, alagebrium, which disassembles AGE by breaking glucose crosslinks, demonstrated encouraging renal effects, did not provide further protective function when tested with an ACE inhibitor [72]. Aminoguanidine and OPB-9195 proved to be potential AGE inhibitors in early trials. Still, sadly, because the end products of the inhibition were revealed to be extremely immunogenic, they also contributed to a decline in renal performance in certain patients due to the accumulation of immune complexes that resulted in glomerulonephritis [73].

The experimental models of DKD have demonstrated improved renal statistics as a result of RAGE suppression, including soluble RAGE and anti-RAGE monoclonal antibodies [68,74]. Since AGE interacts with beta-amyloid to create plaques, azeliragon, as a RAGE inhibitor, has been tested as an option for Alzheimer’s disease therapy in human beings. However, it was unfortunately discovered to cause rapid cognitive deterioration [75]. RAGE also plays a significant part in inflammatory and immune system functioning. Therefore, this potential for negative effects, along with these factors, currently poses obstacles to further study on RAGE inhibitors.

#### 6.1.5. Oxidative Stress

Owing to the unbalanced intracellular formation of free radicals and their scavengers, oxidative stress plays a critical part in the pathophysiology of DN. It has been consistently believed that oxidative stress, which produces and intensifies the metabolic dysregulations of hyperglycemia, is the primary pathogenic mechanism in DM [76]. Through the metabolic process of mitochondria, high glucose in diabetic individuals produces ROS and superoxide anions in tubular epithelial cells and MCs. ROS subsequently stimulates the polyol pathway and AR action, which further stimulates PKC and AGE via the de novo production of DAG. The processes that increase mesangial expansion, thicken the basement membrane, cause impairment of endothelial cells, cause contractions in smooth-muscle cells, and activate TGF-β and cytokines have all been linked to PKC activation in the glomeruli [77]. The ROS damages the podocyte, induces glomerular hypertrophy, and encourages fibrogenesis in the tubules and glomeruli in conjunction with the related oxidized lipids, peptides, nucleic acids, and carbohydrates [78,79].

The nitric oxide synthase (NOS), xanthine oxidase, mitochondria, lipoxygenase, and cytosolic Nox are the origin of ROS production in the DM kidney [80,81]. Regarding diabetic microvascular problems, the consensus was that enhanced metabolism of glucose led to elevated proton gradients, significant electrochemical potential differences, and a higher activity level of the electron transport chain (ETC), all of which facilitated the production of Mt superoxide. Nevertheless, more recent data from metabolomic research indicate a decline in the effectiveness of ETC. Furthermore, it is challenging to measure Mt superoxide, and the results have been contradictory. Some groups have shown a decrease in Mt ROS [26,80]. A certain amount of Mt superoxide may be advantageous and slow down organ malfunction [81,82].

Animal models of DN have persistently shown Nox4 to be elevated, and specific spatial ROS measures are being explored to assess the significance of Mt ROS in DN [83]. It is now established that various DM mediators, like hyperglycemia, AGE, VEGF, TGF-β, endothelin, Ang-II, and aldosterone, can change the function or production of Nox [78]. Numerous Nox4-dependent actions in DN may be attributed to PKC-alpha stimulation via Nox4-mediated mechanisms [84]. Furthermore, Nox4 has been identified to cause fumarate to accumulate by blocking fumarate hydratase. Fumarate is an oncogenic tricarboxylic acid cycle metabolite that has been connected to the activation of TGF-β, hypoxia-inducible factor 1-alpha, and other matrix genes that promote fibrosis [85].

#### 6.1.6. Mitochondrial Dysfunction

The kidney needs a lot of adenosine triphosphate to operate normally because it is a highly metabolic organ with plenty of mitochondria [86]. After the heart, the second highest Mt concentration and oxygen utilization is found in the renal system [87]. Hyperglycemia causes the ETC to become overloaded due to a hike in ROS production. This, in turn, damages DNA and lowers glyceraldehyde-3-phosphate dehydrogenase activity. Therefore, the generation of Mt superoxide is indicative of both physiological oxidative phosphorylation and healthy mitochondria. Stress or a high glucose diet reduces the kidney’s capacity for generating adenosine triphosphate, Mt superoxide, and oxidative phosphorylation. This exacerbates the problem further and causes alterations in Mt shape, reduced peroxisome proliferator-activated receptor-γ coactivator 1-α levels, accelerated cell apoptosis, and escalated Mt division [88,89]. Endothelial NOS activation and declined adenosine monophosphate-activated protein kinase activity are also caused by lower amounts of Mt superoxide. Further, this causes inflammation by impaired vascular function and NF-κB [90].

### 6.2. Hemodynamic Factors

The pathophysiology of DN is also associated with hemodynamic variables involving elevated intraglomerular and systemic BP and varying vasoactive hormone pathway stimulation, like endothelin, RAAS, and urotensin. These changes in hemodynamics activate nuclear TF, different growth factors, and intracellular secondary messengers in conjunction with metabolic pathways, and even independently. In the end, these molecular pathways result in ECM buildup, elevated renal albumin permeability, and exacerbated proteinuria, GMS, and tubulointerstitial fibrosis.

#### 6.2.1. RAAS

Aldosterone, renin, angiotensinogen, Ang-II type 1 receptor, Ang-II type 2 receptor, Ang-I, ACE, and Ang-II comprise RAAS. Every element of the RAAS regulates and upholds both BP and the regular operation of the kidney [90]. Renal disease prognosis is largely influenced by the RAAS, and RAAS blocking has been found to stop the development of CKD, which is marked by reduced proteinuria and systematic kidney functioning [91]. RAAS functioning in DM has been analyzed in-depth, including changes in intraglomerular hemodynamics and alterations in the structure of the tubulo-interstitial and glomerulus [92]. Podocyte cells are known to synthesize a variety of RAAS elements and to display RAAS receptors, including mineralocorticoids, prorenin, and Ang-II receptors. It has been determined that the Ang-II type 1 receptor controls this vital podocyte mechanism [91].

According to the currently available findings, Ang-II is a cytokine that affects the kidneys in a variety of ways [93], encompassing both localized RAS stimulation in the kidney and systemic implications [92]. This implies that Ang-II functioning as a hemodynamic mediator is not limited to its conventional function. Furthermore, mounting research indicates that inflammation, fibrosis, and oxidative stress are among the primary contributors to the onset of disease. The first stage of DN is oxidative stress, which initiates many pathogenic pathways in nearly every type of kidney cell, including tubular cells, endothelial, mesangial, epithelial, and podocyte cells [93]. Overall, it could be said that oxidative stress is a necessary element of cellular destruction, while Ang-II is a “master” component that substantially contributes to renal injury. However, the findings of several trials using therapeutic techniques aimed at Ang-II and oxidative stress are unclear [94,95,96].

Many tissues, including the placenta, heart, uterus, blood arteries, brain, kidneys, and adrenal cortex, possess local renin–angiotensin alongside the circulating RAS. Without the assistance of systemic RAS, kidney cells can manufacture renin, angiotensin receptors, renin receptors, and Ang-II locally, which allows the renal system to sustain elevated intrarenal concentrations of Ang-II [97]. Since the amounts of renal Ang-II in the interstitial space are a thousand times greater than those in the plasma [98], it is thought that intrarenal RAS is primarily responsible for the harm. Indeed, it is well known that elevated glucose stimulates Ang-II and renin generation in MCs [99,100]. There are several implications of intrarenal Ang-II, which may cause kidney damage. Some of these consequences include kidney cellular hypertrophy activation and proliferation, and increased pressure in glomerular capillary permeability, which results in proteinuria, enhancement of inflammation and macrophage infiltration, and ECM and cytokine production [101]. Research has demonstrated that Ang-II blockage has advantages over only decreasing BP. It appears that the blocking impact on RAS can halt the course of the disease at DN, even while systemic renin contents are minimal [102].

#### 6.2.2. Vasoactive Hormones

Following an increasing number of studies, vasoactive hormones act as an essential variable in initiating alterations in the structure and function of the renal parenchyma and vasculature that result in the development of DN. The inner lining of blood vessels is called the endothelium. It carries out several biological functions, such as regulating vascular constriction and preserving the unobstructed arterial flow of blood. By interacting with endothelin A and B receptors, the endothelium releases several vasoactive hormones. Endothelial cells with endothelin B receptors produce prostacyclin and nitric oxide (NO), which help to lower BP. The majority of vascular smooth-muscle cells include endothelin A receptors, which aid in blood vessel constriction. DM induces endothelin over-production in renal tubules’ epithelial cells and glomeruli, which promotes DN [103]. With DM, there is an increased risk of renal illness and an upsurge in ET-1 function [104]. In addition to actively participating in the fibrotic and inflammatory mechanisms of the kidney cells, ET-1 induces proteinuria [105]. A peptide ligand is called urotensin-II. It functions as an agonist for the G-protein-coupled receptor urotensin-II. With strong vasoconstrictive, trophic, and profibrotic effects, this 11-amino acid vasoactive peptide is implicated in heart failure pathogenesis. DM, DN, and renal insufficiency are caused by elevated urotensin plasma concentrations. ROS are produced when urotensin is overexpressed in endothelial cells, which leads to vascular dysfunction in the endothelial system [103].

Renal hemodynamics and the balance of salt and water in the body are two examples of the many cellular and organ functions that NO is known to regulate. It is an endothelium-derived calming agent. NO is formed enzymatically by L-arginine via three NOS isoforms: endothelial (NOS2/endothelial NOS), neuronal (NOS1/nNOS), and inducible (NOS3/iNOS). The kidneys of mammals contain all three of these NOS isoforms. However, NOS1 is more prevalent in the macula densa, medulla, narrow ascending limb of the Henle, and collecting ducts. The glomerular capillaries of the endothelium, proximal tubules, descending vasa recta, renal blood vessels, medullary ascending limb of Henle, and afferent and efferent arterioles all contain NOS2. The collecting duct, medullary ascending limb, and proximal tubules of the S3 segments contain NOS3 [103].

### 6.3. Role of Miscellaneous Signal Transduction Pathways/Receptors/Enzymes/Processes in DN

#### 6.3.1. Wnt Pathway

Protein ligands known as Wnts are a large family that affects several cellular events, such as polarity maintenance within cells, control of cell fate, and embryonic commencement [106]. The glycoproteins in the Wnt pathway are high in cysteines. The Wnt pathway signal transduction is essential for oncogenesis and embryogenesis, and it also functions as a regulator for several disorders, including T2DM, prostate, breast, and brain malignancies. Although it is comparatively inactive in normal kidneys, Wnt canonical signaling is increased with renal damage [107,108]. According to Guo et al. [109], the Wnt canonical pathway has been identified as a prominent controller in the progression of DN. The two routes that constitute the Wnt signaling pathway are the “canonical β-catenin (β-Cat) dependent pathway” and the “non-canonical β-Cat independent pathway”. Common canonical pathway activators involve “Wnt1, Wnt2, Wnt3, Wnt3a, Wnt8a, Wnt8b, Wnt10a, and Wnt10b”. Non-canonical pathway activators involve “Wnt4, Wnt5a, Wnt5b, Wnt6, Wnt7a, Wnt7b, and Wnt11” [110].

**Figure 4:**
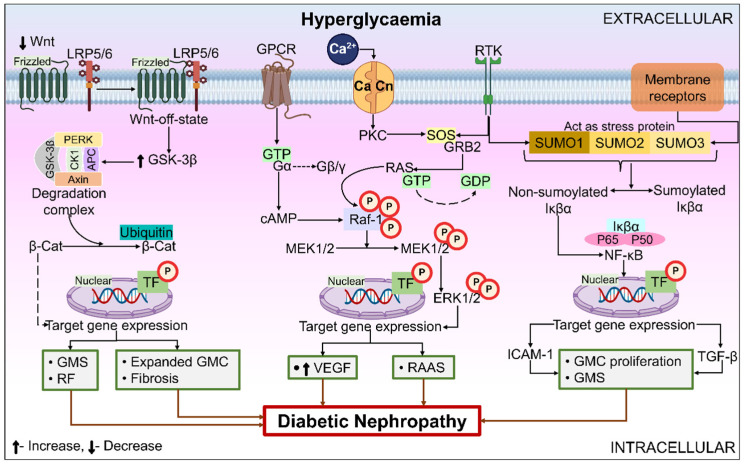
An illustration of the roles that the Wnt, extracellular signal-regulated kinase (ERK), and NF-κB play in the pathophysiology of DN. When a hyperglycemic condition is delivered to GMC, the expression of Wnt4 and Wnt5a mRNA is blocked, which raises the activity of GSK-3β, resulting in the overproduction of ECM, causing DN. MEK1/2 causes ERK1/2 and RAS activation, which induces kidney cell destruction, promoting the occurrence of DN by inducing regional inflammation and promoting the growth and hypertrophy of the arteries and GMC. In podocytes, ERK activation causes upregulated VEGF mRNA and protein expression, further promoting an upsurge in glomerular hypertrophy, hyperfiltration, and albuminuria. ICAM-1 and TGF-β1 are expressed when nuclear translocation of hyperglycemia-activated NF-κB occurs. As a result, prolonged and increased inflammation, along with excessive FN synthesis, encourages GMC proliferation and hastens GMS, causing DN. Abbreviations: Wnt, wingless-related integration site; GSK-3β, glycogen synthase kinase-3β; PERK, protein kinase R-like endoplasmic reticulum kinase; CK1, casein kinase-1; APC, adenomatosis polyposis coli; β-Cat, β-catenin; TF, transcription factor; GMS, glomerulosclerosis; RF, renal fibrosis; GMC, glomerular mesangial cells; GPCR, G protein-coupled receptors; GTP, guanosine triphosphate; cAMP, cyclic adenosine monophosphate; MEK1/2, MAPK/ERK kinase 1/2; ICAM-1, intercellular adhesion molecule-1; Raf-1, rapidly accelerated fibrosarcoma-1; VEGF, vascular endothelial growth factor; RAAS, renin–angiotensin–aldosterone system; RAS, renin–angiotensin system; ERK1/2, extracellular signal-regulated kinases 1 and 2; SUMO1 and SUMO2/3, small ubiquitin-related modifier1; SOS, son of sevenless; RTK, receptor tyrosine kinase; LRP5/6, Low-density lipoprotein receptor-related protein 5/6; GDP, guanosine diphosphate; GRB2, growth factor receptor 2; NF-κB, nuclear factor-κB; TGF-β, transforming growth factor-β.

The inter-species mechanism of conservation of the canonical Wnt signaling pathway is remarkable. The human and mouse genomes contain more than eighteen different types of Wnt-related proteins. Glycogen synthase kinase-3 (GSK-3) enzyme activity is increased by the down-regulation of Wnt and intracellular signaling stimulation in glomerular mesenchymal cells amid excessive glucose stress, which is achieved by inhibiting the production of Wnt4 and Wnt5a messenger RNA (mRNA). A β-Cat degradation complex comprising protein kinase R-like endoplasmic reticulum kinase, GSK-3, axin, casein kinase-1, and adenomatosis polyposis coli promotes the N-terminal phosphorylation of catenin. Proteases break down the β-Cat as a result, keeping the minimal protein levels in cells. Down-regulated nuclear β-Cat promotes the expression of TGF and fibronectin genes by preventing the synthesis of TF-β-Cat. ECM gene expression increases as a result, and renal fibrosis (RF), GMS, expanded glomerular mesangial cells (GMC), and fibrosis are the outcomes of extensive ECM buildup [111]. Figure 4 depicts the Wnt pathway’s contribution to the pathophysiology of DN.

#### 6.3.2. Extracellular Signal-Regulated Kinase (ERK) Signaling Pathway

The ERK pathway is the main signaling pathway that maintains cellular proliferation, differentiation, and death. Research has clearly stated that, in DN, ERK possesses a crucial function in coordinating gene transcription, which is involved in a variety of response mechanisms in the cell. ERK signaling in the pathology of DN is presented in Figure 4. Different kidney cells, including podocytes, MCs, and growth factors, can activate the ERK pathway. The threonine and tyrosine residues of different MAPK isoforms are phosphorylated by activated Raf-1 kinase, which later activates the MAPK/ERK kinase 1/2 that activates ERK-1/2 and eventually activates RAAS, under the direction of hemodynamic and metabolic factors. For instance, it is well known that, when the RAAS elements are engaged, Ang-II may cause renal cell injury. Numerous mechanisms contribute to this: RF stimulation, kidney damage brought on by pressure, chemokine generation, and sensitization of TGF-β and ROS, which induce regional inflammation and promote MCs proliferation, elimination of albumin in the kidneys, hyperfiltration, and glomerular hypertrophy in diabetic rats [112]. In DN, it is an essential signaling route. More studies are needed to comprehend the ERK mechanisms and pinpoint particular target proteins and medications to cure DN.

#### 6.3.3. NF-κB

Transcription factor NF-κB is activated via different inflammatory components in DN, including AGE, hyperglycemia, and mechanical stress. It is among the fundamental elements implicated in the inflammatory mechanisms of DN, as presented in Figure 4. Moreover, DN kidney damage is facilitated by NF-κB regulation of chemokines, inflammatory cytokines, and cell adhesion proteins [113]. NF-κB’s persistent presence in cells, even in an inactive state, makes it a significant “first respondent” to DN. Because of this, the pathways implicated can activate more quickly as the synthesis of proteins by these TFs is not necessary for the stimulation of the pathways. The Janus kinase (JAK)–signal transducers and activation of transcription (STAT) pathway is amongst the primary pathways that react to and transduce inflammatory signaling. Important processes governing cell stimulation, proliferation, recruitment, migration, and differentiation can be triggered by cytokines and hyperglycemia [50]. There’s growing evidence that JAK-STAT is essential to the pathophysiology of DN. JAK-STAT has been identified to be elevated in glomerular cells in early DN subjects. Similarly, as the disease progresses, the expression of different JAK and STAT isoforms in the tubulointerstitial space rises and has an inverse relationship with the estimated GFR. JAK-STAT triggers NF-κB activation, a crucial TF in the inflammatory pathways of DN. NF-κB triggers the transcription of adhesion molecules, chemokines, and proinflammatory cytokines after it is activated [113].

#### 6.3.4. JAK-STAT

**Figure 5:**
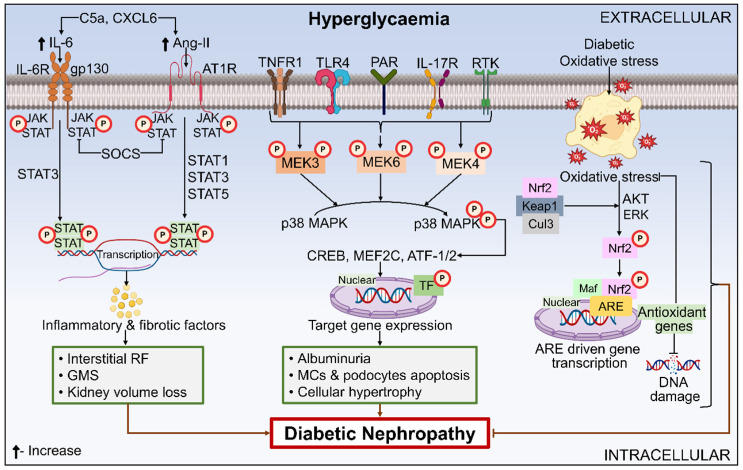
An illustration displaying the role of JAK-STAT, MAPK, and Nrf2 in the pathogenesis of DN. JAK/STAT signaling is initiated by interleukin-6 and ang II, which further triggers the immune system, fibrosis, aging, and autophagy, and aggravates signs of kidney injury, including GMS, interstitial RF, and renal volume loss. Hyperglycemia causes a rise in MAP kinase kinase3/6/4 activity and mRNA expression, which activates transcription factors. These transcription factors then regulate FN transcription and protein buildup, ultimately resulting in DN by inducing MCs and podocyte apoptosis, as well as cellular hypertrophy. In its natural state, Nrf2 localizes to the cytoplasm where it binds to Keap1, causing the proteasome to ubiquitinate and degrade Nrf2. However, in the presence of diabetic oxidative stress, Nrf2 separates from Keap1, and this degradation is prevented. Together with Maf proteins, Nrf2 builds a heterodimer, where it binds to the ARE sequence to start the transcription of various Nrf2 downstream genes that prevent oxidative damage or stress. Abbreviations: IL, interleukin; Ang-II, angiotensin-II; gp130, glycoprotein 130; AT1R, angiotensin II type 1 receptor; JAK, Janus kinase; STAT, signal transducer and activator of transcription; RF, renal fibrosis; GMS, glomerulosclerosis; RTK, receptor tyrosine kinase; MEK, MAPK/ERK kinase; MAPK, mitogen-activated protein kinase; TF, transcription factor; MCs, mesangial cells; AKT, Ak strain transforming; ERK, extracellular signal-regulated kinase; SOCS, suppressors of cytokine signaling; ATF-1, activating transcription factor-1; MEF2C, myocyte enhancer factor-2C; CXCL6, C-X-C motif chemokine ligand 6; gp130, glycoprotein 130; CREB, cAMP response element binding protein; TNFR1, tumor necrosis factor receptor 1; TLR4, toll-like receptor 4; PAR, protease-activated receptor; Cul3, cullin 3; ARE, antioxidant response element; Nrf2, nuclear factor erythroid 2-related factor 2; Keap1, Kelch-like ECH-associated protein 1.

A set of intracellular signaling molecules known as the JAK-STAT pathway activates target genes to provide mechanistic action for several hormones, growth factors, and cytokines. Through the JAK-STAT pathway, multiple variables, such as fibrosis, epithelial-to-mesenchymal transition, aging, RAS, autophagy, inflammatory response, and the immune system, accelerate the DN mechanism, as illustrated in Figure 5. In diabetic renal tissues, these factors also lead to an upsurge in JAK and STAT expression [114]. Recently, a significant elevation in the number of JAK-STAT elements has been identified in DN, based on a study of the transcription patterns of tissue samples from the kidneys of individuals with the disease [54]. The addition of a JAK1/JAK2 inhibitor largely restored the primary phenotypic alterations associated with DN, as demonstrated by animal models where overexpression of the JAK pathway exacerbated the functional and clinical aspects of DN [115].

According to Berthier et al. [116], there is a considerable hike in the levels of STAT1/3, JAK1-3, mRNA, and protein in the glomeruli and tubulointerstitium of DN individuals. This is due to JAK2-STAT signal stimulation, which in turn, causes glomerular sclerosis and tubulointerstitial fibrosis. JAK2 expression was determined to be elevated in the tubulointerstitial area of progressive DN subjects, in contrast to the glomeruli of early DN patients. This finding is captivating, as it suggests that tubulointerstitial pathological modifications in DN follow the pathogenic transformations of glomerular damage that progress naturally [54].

Research has demonstrated that T1DM C57BL/6 mice exhibit elevated STAT3 phosphorylation in their tubular cells of the kidney, while in treatment with the STAT3 inhibitor S3I-201, diabetic mice showed improvements in inflammatory response, RF, and kidney functioning [117]. Akita diabetic podocyte JAK2 mice exhibit an overexpression of JAK2 in the glomerular and tubulointerstitial cells and podocytes, which further contributes to DN-associated pathological alterations such as mesangial dilatation, RF, GMS, and albuminuria [115]. Albuminuria was shown to decline in a newly reported Phase 2 trial with the JAK1/JAK2 inhibitor baricitinib, which was administered orally [118]. To define the application of JAK inhibitors in the future, additional trials are necessary to ascertain how these drugs affect the progression of DN. JAK1 and JAK2 inhibitor-treated diabetic mice revealed that STAT3-dependent genes, like TGF-β, Notch1, and C-C chemokine receptor type 2, which are the main drivers of diabetic neuropathic pain, express less when treated [115].

#### 6.3.5. MAPK Signaling Pathway

The MAPK signaling pathway comprises a group of Ser/Thr protein kinases. They cause the activation of an amplifying cascade, which controls the transformation of signal transduction into cellular transduction and has diverse effects on the cells. MAP3K, MAPKK, and MAPK are three kinases that comprise the heart of the MAPK pathway. After being phosphorylated, MAP3K triggers MAPKK, which in turn, triggers MAPK. c-Jun N-terminal kinase 1/2/3, ERK1/2, ERK5, and p38MAPK are the four sub-families. Recent research has demonstrated that modifications to the MAPK pathway impact the development and course of DN. Elevated blood sugar levels have been linked to phosphorylation and p38MAPK signaling pathway activation. This, in turn, promotes MCs’ fibronectin production, which stops the advancement of DKD and thickens the glomerular basal membrane. Hyperglycemic stimulation of RAAS via the p38MAPK pathway leads to renal impairment in DM patients [119]. Sulodexide, a sulphated glycosaminoglycan, and seintib, an inhibitor of MAP-3K5, both lessen kidney damage in T1DM and T2DM by focusing on p38MAPK [120]. The role of MAPK in DN has been depicted in Figure 5.

#### 6.3.6. Apoptosis Signal-Regulating Kinase (ASK) 1

ASK1 is an upstream kinase in the MAPK pathway that accelerates the development of DKD and causes RF when it is triggered by inflammation and oxidative stress [121]. Renal biopsy samples from DKD patients have been shown to exhibit higher ASK1 expression. ASK1 small-molecule inhibitor GS-444217 has recently been the subject of preclinical animal investigations that show improved DKD histopathological characteristics, decreased albuminuria, and a decreased drop in estimated GFR. Combining ASK1 inhibition with an ACE inhibitor showed better renal-protection than either drug alone in DM mice, which may have clinical significance [122]. ASK1 inhibitor clinical studies have commenced, and encouraging antifibrotic outcomes have been observed in non-alcoholic steatohepatitis. Hence, it will be fascinating to observe if the implications of DKD in animal models extend to humans.

#### 6.3.7. Nox

Numerous renal cells express Nox isozymes, including tubule cells (Nox4 and Nox1), glomeruli (Nox1, Nox2, Nox4, and Nox5), endothelial cells (Nox4), and smooth-muscle cells (Nox1). The RAAS pathway, PKC, AGE production, and TGF-induced fibrosis are the hallmarks of DN development. Hyperglycemia causes the body to manufacture more AGE, which are ligands for RAGE and indicate inflammation. Interleukin-6 and tumor necrosis factor-α are synthesized when AGEs activate signaling networks and increase NF-κB activity. MC hypertrophy, which is a cause of ECM accumulation, hypertrophy, and glomerular basement membrane thickening, is triggered by AGE through the activation of PKC and VEGF production, which in turn, stimulates Nox isoenzymes. Following extensive screening, the lead compounds were discovered as two Nox inhibitors, APX-115 and GKT136901, which prevented DN [123].

#### 6.3.8. Nrf2

One of the most significant mediators of oxidative stress is the TF- Nrf2. Systemic oxidative overload is attenuated by antioxidant cytoprotective genes whose expression is regulated by Nrf2 [52]. Under a normal body state, Nrf2 interacts with its inhibitor, Keap1, to become continuously ubiquitinated and destroyed by proteasomes. By opposing Keap1 interactions with Nrf2, electrophilic chemicals or oxidative stress stabilize Nrf2. As a result, the nuclear translocation of Nrf2 occurs, wherein it attaches itself to components that respond to antioxidants. Consequently, there is a rise in the gene transcription that codes for detoxifying enzymes and antioxidants, including heme oxygenase-1, glutathione S-transferase, quinine oxidoreductase 1, and γ-glutamyl cysteine synthetase. Instead of depending only on one antioxidant enzyme, Nrf2 activization leads to an overexpression of various antioxidant enzymes, which is the most alluring aspect of considering the Nrf2/Keap1 pathway as a potential lead target [124]. Evidence for Nrf2’s protective role in renal disease has been shown by the considerably more severe kidney injury sustained by Nrf2−/− mice in contrast to wild-type mice [125]. The role of Nrf2 in DN has been presented in Figure 5.

#### 6.3.9. Autophagy

When proteolytic enzymes found in lysosomes assist in the breakdown and recycling of proteins and organelles as a source of energy, the process known as autophagy takes place in cells. Autophagy is pivotal in the natural elimination of aging and damaged cellular structures, enhancing tissue homeostasis and cell viability [126]. The enhanced lifespan associated with caloric restriction is assumed to be due to the heightened autophagy that occurs in famine states for energy salvage [127]. However, uncontrolled autophagy can also be harmful because it can cause apoptosis [126]. DN pathophysiology is linked to impaired autophagy. Diabetes-related hyperglycemia prevents autophagy, which prevents the clearance of the contents of cells destroyed by oxidative stress or AGE. This can lead to kidney cell injury and RF through the control of TGF-β [128]. Thus, the question of whether autophagy stimulation is advantageous has been investigated in recent studies. The mammalian target of the rapamycin pathway has been linked to the pathophysiology of DN and autophagy regulation. As a result, there has been interest in using rapamycin to activate autophagy through the mammalian targets of rapamycin inhibition as a therapeutic strategy. In fact, using this strategy over brief periods of time has demonstrated some encouraging effects on the histological alterations of DN in animal models [129]. Future research on alternative autophagy-promoting mechanisms, including the silent information regulator T1 and adenosine monophosphate-activated protein kinase, may be beneficial for renoprotection [126]. Though there have not been any fruitful clinical trials until now, there is still ongoing research toward the application of medicines that target the autophagy pathways in DN.

#### 6.3.10. Fibrosis

RF is frequently the final response to the damage, which causes DN to advance and ultimately results in ESRD. As part of their biological effect, several recently highlighted treatment approaches, such as RF, are lessened by inhibition of RAS [130]. The main cause of tissue fibrosis in DN and other types of CKD is TGF-β. Upsurged ECM synthesis, the suppression of ECM breakdown, and myofibroblast stimulation are all caused by TGB-β activation [131]. Nonetheless, because TGF-β plays crucial roles in immunoregulation, it might be challenging to target the protein directly. TGF-β inhibition in DN in animal models has been shown to be useful in reducing fibrotic transformation [132]. Recently, in an unfortunate turn of events, clinical research in phase 2 included an anti-TGF-β monoclonal antibody, which was abruptly ended because of significantly lower efficiency [133]. Since TGF-β is a downstream effector that contributes to the fibrosis process and induces connective TGF, the suppression of connective TGF is a further antifibrotic strategy that is still being researched. Its involvement in the etiology of DKD has been shown in animal models of connective TGF suppression [134].

#### 6.3.11. Epigenetics

By modifying which regions of DNA coding are available for transcription and translation via processes including histone post-translational changes, DNA methylation, and microRNA, epigenetics addresses the regulation of gene expression. Normal cellular differentiation and the onset of diseases like DKD can both be attributed to epigenetic alterations caused by extrinsic and internal stimuli, which can change the phenotype of the cell [135]. One mechanism of epigenetic regulation that has been linked to DKD is DNA methylation. Numerous investigations have shown that renal cells from people with DKD have different methylation profiles than those of patients without the disease. As a matter of fact, it has been indicated that these variations in DNA methylation act as biomarkers for predicting the state and course of disease [136]. To affect cell development and the production of specialized tissues like the glomerulus, histone proteins regulate gene expression. An expanding corpus of research links significant epigenetic modifications in histone methylation to DM complications, such as DKD. The function of specific histone methyltransferases in DM problems, including a reason for the clinical phenomena of metabolic memory, was first found by seminal investigations [137]. Moreover, DKD has been directly linked to the enzyme Set7, which methylates histone 3 lysine 4 [138].

## 7. Role of Blood Stasis in DN and Its Molecular Mechanism

According to traditional Chinese medicine, blood stasis, also known as Xueyu in Chinese, is a clinical state brought on by an obstruction or stoppage of blood flow and is thought to be the cause or outcome of multiple chronic medical conditions [139]. The symptoms of blood stasis include a hard, fixed abdominal mass, cyanosis of the lips and skin, scaly skin, a subcutaneous hematoma or purpura, dryness in the mouth, and sharp pain in a fixed area that is exacerbated by pressure and gets worse at night [140]. The primary clinical signs of DN are increasing renal impairment, edema, and proteinuria. Traditional medicine views blood stasis-induced collateral blockage as the essential pathogenesis of DN. The most effective medications for treatment are those that increase blood flow to get rid of blood stasis and dredge collateral. DN has often been treated by generating prescriptions for removing collateral and dispersing blood stasis [141,142]. Under normal circumstances, the kidney’s collaterals help the kidney get rid of extra water and waste products from metabolism in the blood. When kidney collateral stasis develops in pathological situations, it may indicate a number of glomerular issues, including lumen stenosis, renal balloon wall disintegration, mesangial matrix increases, vascular necrosis, or even blockage. VEGF is the most potent vascular permeability factor that has an impact on the glomerular filtration barrier. Massive proteinuria develops after the glomerular extracellular matrix builds up, the basement membrane thickens, glomerular sclerosis and renal interstitial fibrosis proceed, and the extravasation of blood components is worse. In traditional Chinese medicine, this is quite similar to the pathological condition where “blood does not follow the meridian, overflowing outside the pulse, and interferes with the kidney collaterals” [143,144]. Based on years of clinical research and its findings, Professor Zheng Nan concluded that DKD is a chronic disease of yin and yang deficiency, culminating in many pathologic outcomes, such as phlegm, dampness, and blood stasis. The primary pathological mechanism of DKD may be the internal buildup of toxic pathogenic factors that affect the circulation of Qi-blood (“Qi” refers to the life force or vital energy that runs through the body). This accumulation may be caused by the movement of bodily fluids, which damages renal collaterals, injures the kidneys, and depletes kidney Qi [143].

## 8. Conclusions

This review outlines the current advancements in the biomolecular mechanisms behind the pathogenesis of DN, with the conclusion that the pathophysiology of DN is indubitably multifaceted and complex in nature. Both T1DM and T2DM share a common DN pathologic mechanism that includes growth factors such as hemodynamic, metabolic, inflammatory, and fibrotic elements. Moreover, the literature indicates a number of risk factors for DN, such as smoking, a lack of physical activity, uncontrolled arterial pressure, a lengthy history of DM, and inadequate blood sugar control. To provide better renoprotection in diabetes, combined strategies targeting a variety of hemodynamic and metabolic mechanisms are probably required. Additionally, by fully comprehending the fundamental pathologic mechanisms of DN, the effective markers employed in its development need to be determined to measure the effectiveness of the therapy. It is envisaged that other potential targets and, eventually, novel medicines that can block numerous harmful pathways could be discovered, considering the recent findings of important areas of interactions among glucose and BP-dependent pathways in DN. Although there have been significant advances, more work is still needed to construct an understandable overview of the biomolecular mechanisms responsible for the pathogenesis of DN, which will help in developing safe and potent treatments for DN.
